# Early Interceptive Management of Bilateral Canine Impaction With Anterior Crossbite Using Double Extraction and a 2 × 4 Appliance

**DOI:** 10.1155/crid/1080772

**Published:** 2026-07-09

**Authors:** Deemah Alhamawi, Lena Gary, Raniah Baakdah

**Affiliations:** ^1^ Ministry of National Guard for Health Affairs (MNGHA), Department of Pediatric Dentistry, King Abdullah Specialist Children′s Hospital (KASCH), Jeddah, Saudi Arabia; ^2^ Ministry of Health, Diriyah Hospital, Riyadh, Saudi Arabia, moh.gov.sa

**Keywords:** CBCT, interceptive orthodontics, maxillary canine impaction, mixed dentition

## Abstract

**Background:**

Maxillary canine impaction is a common developmental eruption disturbance that may lead to root resorption of adjacent incisors, crowding, and prolonged treatment if not identified early. Early recognition during mixed dentition allows interceptive measures that may guide eruption and reduce the need for surgical exposure.

**Case Presentation:**

A 9‐year‐old Saudi girl presented with an anterior crossbite, functional mandibular shift, poor oral hygiene, and multiple carious deciduous molars. Clinical examination showed absence of palpable maxillary canine bulges, and panoramic radiography with CBCT confirmed bilateral palatal impaction of the maxillary permanent canines with S‐sector 3, *α*‐angle 60°, d‐distance about 22 mm, and close proximity to the lateral incisor roots without resorption. Preventive and restorative care were completed first, followed by the extraction of deciduous canines and first deciduous molars (53, 54, 63, and 64) and the placement of a maxillary 2 × 4 fixed appliance to correct the anterior crossbite and maintain space. Over 14 months, both permanent canines erupted spontaneously into favorable positions without lateral incisor root resorption, and the mandibular shift resolved.

**Conclusion:**

In this mixed‐dentition patient with bilateral palatal canine impaction and moderate radiographic risk indicators, a double extraction protocol combined with a simple 2 × 4 appliance enabled successful nonsurgical eruption guidance and simultaneous correction of anterior crossbite.

## 1. Background

Maxillary canine impaction is the second most frequent dental impaction after third molars and may result in root resorption of adjacent teeth, crowding, arch‐length loss, and aesthetic or functional problems if untreated [1–3]. Early clinical signs include absence of a canine bulge, delayed eruption, asymmetrical exfoliation, and retained deciduous canines, whereas radiographic indices such as sector position, *α*‐angle, and vertical position help estimate severity and prognosis [4–7]. This report describes the interceptive management of bilateral palatal maxillary canine impaction in a mixed‐dentition child using double extraction and a 2 × 4 appliance, with concurrent correction of anterior crossbite and functional shift.

## 2. Case Presentation

A 9‐year‐old Saudi female with no relevant medical history presented for dental care because of posterior caries. Extraoral examination showed a mild mandibular shift with normal temporomandibular joint function and jaw opening. Teeth are identified using the FDI World Dental Federation two‐digit system. Intraoral examination showed poor oral hygiene, multiple carious deciduous molars, anterior crossbite involving Teeth 11 and 12 with a functional mandibular shift to the right, and absence of palpable permanent canine bulges. Molar‐incisor hypomineralization affected Teeth 31 (Code 2) and 46 (Code 3), which influenced preventive and restorative planning.

Bitewing and periapical radiographs were obtained to assess caries and restorability of the deciduous molars. Panoramic radiography showed ectopic bilateral maxillary permanent canines with S‐sector 3, *α*‐angle 60°, d‐distance about 22 mm, V‐height less than half of the lateral incisor root length, and apices above the first premolar roots. CBCT confirmed bilateral palatal impaction and close proximity to the lateral incisor roots without resorption. Study models and Moyers space analysis showed mild maxillary crowding.

**Figure 1:**
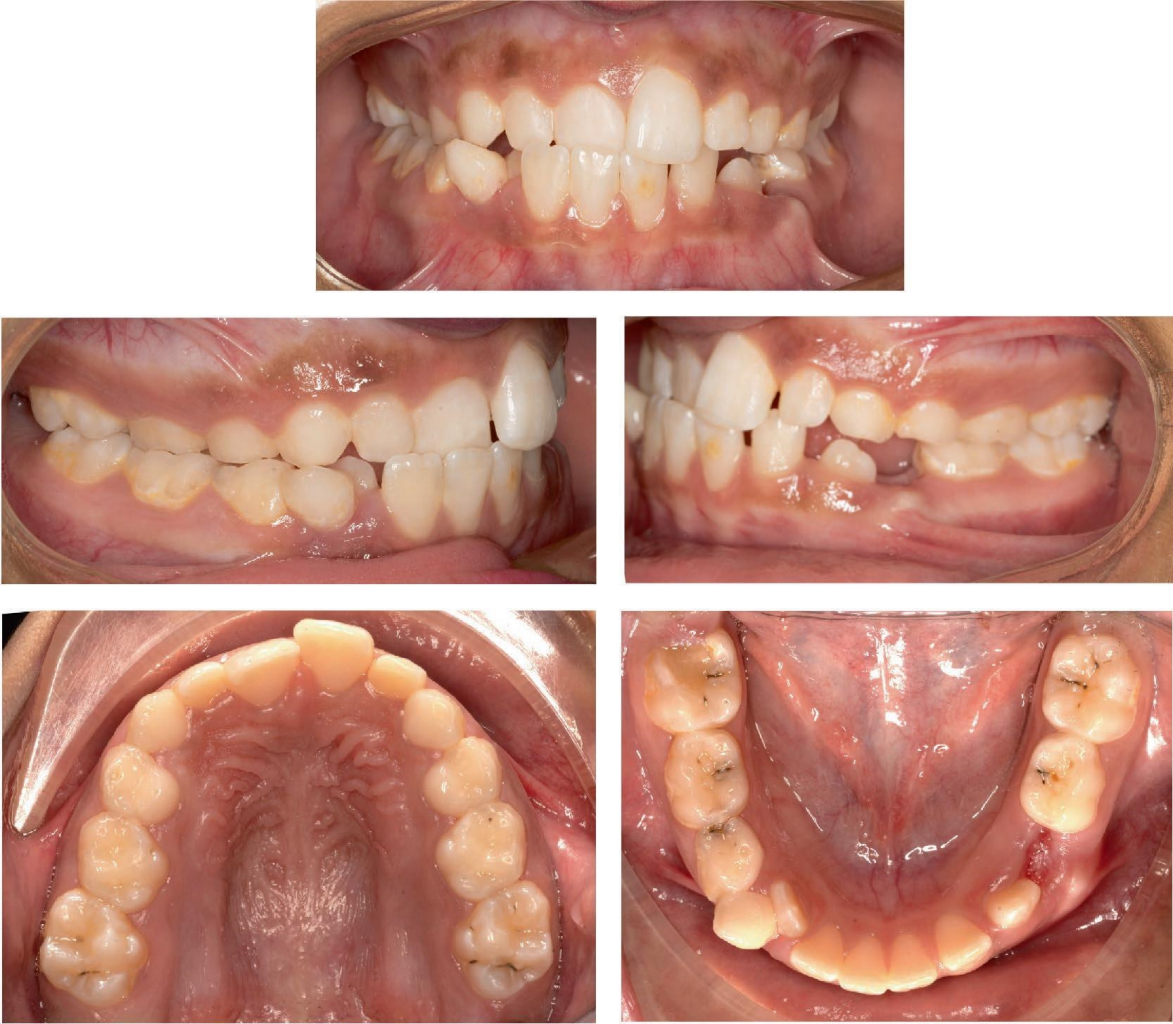
Pretreatment clinical documentation.

**Figure 2:**
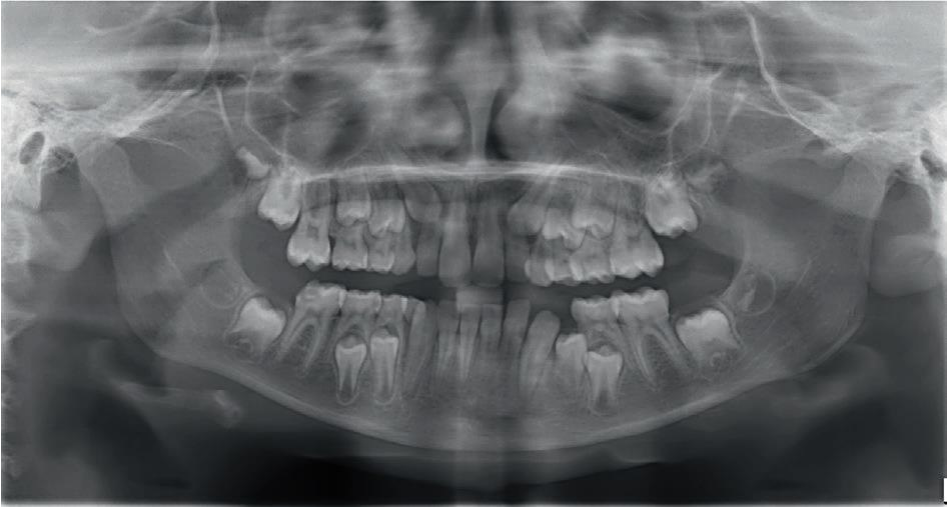
Pretreatment radiographic assessment panoramic radiograph.

Based on the clinical and radiographic findings, both canines were considered to be moderately impacted, with a realistic potential for interceptive management. The objective of treatment was to create localized space, reduce the risk of lateral incisor root resorption, promote spontaneous canine eruption, and simultaneously correct the anterior crossbite. (Documentation file: Figures 1, 2).

### 2.1. Therapeutic Intervention

Treatment was completed in sequential preventive, restorative, orthodontic, and maintenance phases. Preventive care includes oral prophylaxis, oral hygiene instruction, and dietary counseling. Restorative care includes stainless steel crowns and composite restorations for the carious deciduous molars, with attention to the MIH‐affected teeth to reduce hypersensitivity and support long‐term function.

**Figure 3:**
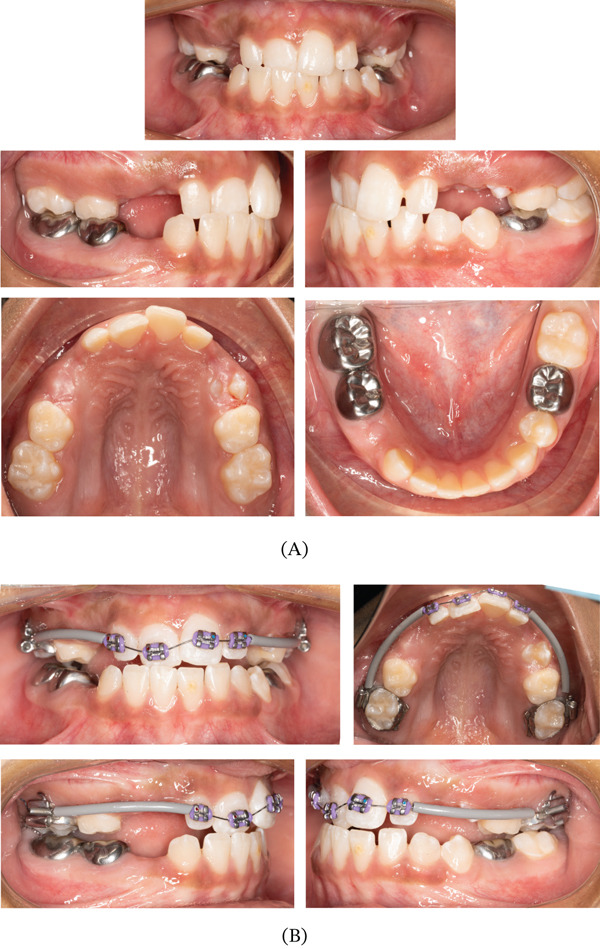
Treatment progression sequence. (A) Postextraction clinical view after removal of primary canines and first molars. (B) 2 × 4 appliance placement with initial 0.014 ^″^ NiTi archwire and GIC bite blocks.

Interceptive orthodontic treatment included the extraction of deciduous canines and first deciduous molars (53, 54, 63, and 64) to accelerate eruption and create space. This double extraction approach was selected because the patient had mild crowding, adequate transverse width, a Class I molar relationship, moderate impaction severity, and a need for more localized space creation adjacent to the lateral incisors. A maxillary 2 × 4 appliance was then placed using bands on Teeth 16 and 26 and brackets on Teeth 12, 11, 21, and 22, with sequential archwire progression and occlusal build‐ups on Teeth 36 and 46 to facilitate correction of the anterior crossbite. (Documentation file: Figure 3A,B).

### 2.2. Follow‐Up and Outcomes

**Figure 4:**
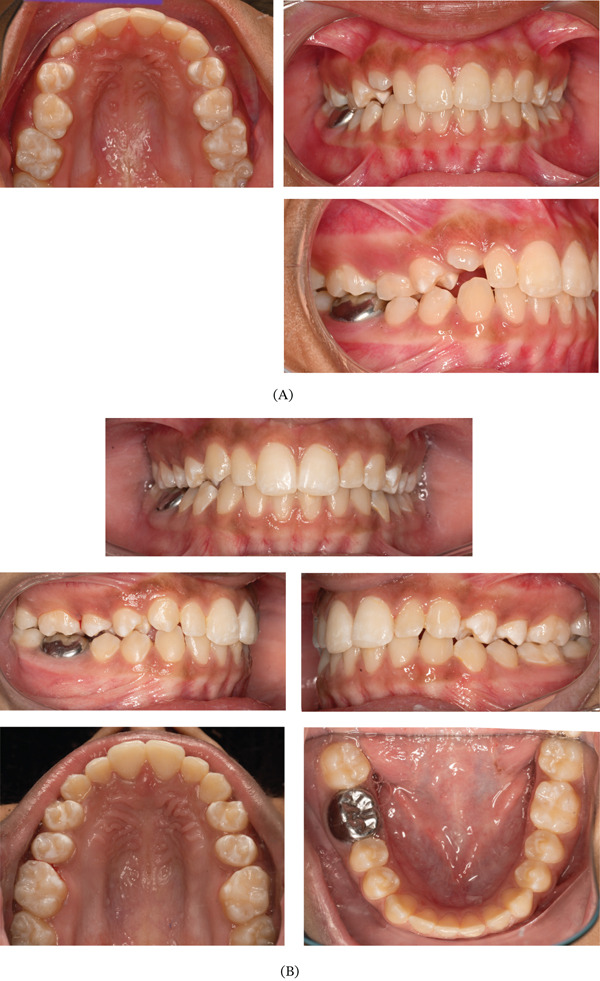
Posttreatment clinical outcomes. (A) 14‐month intraoral view showing erupted canines #13 (B). 24‐month views showing complete bilateral canine eruption.

**Figure 5:**
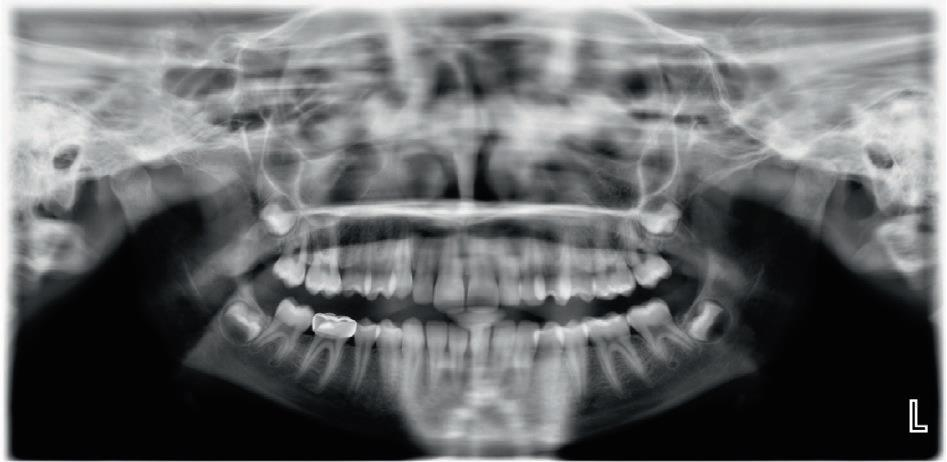
Posttreatment radiographic documentation: 24‐month panoramic radiograph.

Progress was monitored clinically and radiographically during follow‐up visits (Documentation file: Figures 4A,B and 5). By Month 14, both maxillary permanent canines had erupted spontaneously into ideal occlusal positions, the anterior crossbite had resolved, the functional mandibular shift had been eliminated, and periodontal tissues were healthy. Radiographic follow‐up confirmed normal canine development and the absence of root resorption in Teeth 12 and 22.

From the family′s perspective, the extraction of four deciduous teeth initially caused anxiety, and the child experienced a short adaptation period with mild discomfort and temporary speech difficulty after placement of the 2 × 4 appliance. The family reported satisfaction with the treatment duration, avoidance of surgical exposure, and improvement in the child′s smile and self‐confidence.

## 3. Discussion

This case illustrates the value of systematic clinical screening and early radiographic assessment in mixed dentition when canine bulges are absent, and eruption appears delayed [4, 5]. The combination of S‐sector 3, *α*‐angle 60°, d‐distance about 22 mm, and proximity to the lateral incisor roots indicated a moderate impaction severity, which is a clinically significant but interceptable condition. The absence of root resorption throughout follow‐up supports the benefit of early intervention before irreversible damage occurs. [6, 7]

Evidence from metaregression and clinical guidelines supports the interceptive extraction of the deciduous canine alone to normalize eruption in approximately 62%–91% of palatally displaced maxillary canines, although success is influenced by canine overlap and patient age, [8] and reduces the need for surgical exposure, often complemented by space maintenance or simple fixed appliances when indicated [9–11]. The rationale in this patient favored adding the extraction of the first deciduous molar to create more localized space and potentially shorten the time spent in a resorption‐risk position [12, 13]. Nevertheless, recent pooled analyses of ectopic case management using single‐ or double‐extraction protocols reported broadly comparable overall successful eruption into the arch, even double extraction yields superior radiographic positional changes [14]. The 2 × 4 orthodontic appliance efficiently addressed the concurrent anterior crossbite while providing controlled force application to prevent damage to the lateral incisor roots, and it was also used for space management to achieve successful outcomes [15, 16].

Based on current evidence, treatment duration for impacted canines is generally lengthy, particularly for palatal or mesially positioned canines and those with greater inclinations, with palatal canines often requiring roughly twice as long to emerge as buccal canines [17, 18]. Multiple factors likely contributed to the relatively favorable and efficient outcome, including early detection during the optimal mixed‐dentition window [1, 3, 4], comprehensive assessment to refine diagnosis and planning [19], appropriate case severity selection with favorable prognostic indicators [6], a sequential treatment strategy that progressed logically from space creation to controlled orthodontic guidance [12], and excellent patient cooperation throughout the treatment period.

The coexistence of MIH added a restorative and preventive dimension to treatment planning. Management addressed both eruption guidance and preservation of function in Teeth 31 and 46 through restoration, symptom control, and planned long‐term review.

## 4. Patient Perspective

From the family′s perspective, proceeding with the extraction of four deciduous teeth initially caused anxiety about pain, chewing, and the child′s appearance, but a detailed explanation of the plan and its aim to minimize visits and overall burden was reassuring. The child ultimately found the extractions easier than expected, adapted quickly to soft foods, and did not experience persistent pain or chewing difficulties; she was briefly self‐conscious about the fixed appliance but became comfortable once it was normalized by peers and teachers, and as the anterior crossbite and canine eruption visibly improved. The parent reported that the 2 × 4 appliance required only about a week of adaptation with mild discomfort and transient speech changes before daily function and oral hygiene returned to normal, and the family expressed satisfaction with the treatment duration, avoidance of surgical exposure, and the improvement in the child′s smile and self‐confidence; they also understood that, because of coexisting MIH, ongoing monitoring and possible future restorative or endodontic treatment for affected molars might be needed.

This report is limited by its single‐case design and lack of long‐term posteruption follow‐up. However, it provides a practical example of coordinated pediatric dental and interceptive orthodontic care supported by a supplementary timeline and radiographic documentation.

## 5. Conclusions

Systematic clinical screening in mixed dentition can support early suspicion of maxillary canine impaction when canine bulges are absent, and eruption is delayed. In selected pediatric patients with bilateral palatal impaction and moderate radiographic risk factors, interceptive extraction of deciduous canines and first deciduous molars may provide sufficient space for spontaneous eruption without the need for surgical exposure. Concurrent correction of anterior crossbite and functional mandibular shift can be integrated into the same interceptive phase to improve function and reduce overall treatment complexity.

**Nomenclature**
2 × 4: Two‐by‐four orthodontic appliance
CBCT: Cone‐beam computed tomography
MIH: Molar‐incisor hypomineralization
NiTi: Nickel‐titanium

## Author Contributions

Deemah Alhamawi: patient assessment, restorative, and interceptive orthodontic intervention treatment, data collection, and manuscript drafting. Lena Gary: patient assessment and 2 × 4 orthodontic intervention treatment, and manuscript drafting. Raniah Baakdah (corresponding author): treatment planning and supervision, case study conception, manuscript editing, and review.

## Funding

No funding was received for this manuscript.

## Disclosure

All authors read and approved the final manuscript.

## Ethics Statement

This case report was conducted in accordance with the Declaration of Helsinki and approved by the Institutional Review Board of King Abdullah International Medical Research Center (KAIMRC) (Protocol [H‐01‐R‐005]). Written informed consent was obtained from the patient′s parent/guardian for participation in this study.

## Consent

Written informed consent was obtained from the patient′s parent/guardian for publication of this case report and any accompanying images. A copy of the written consent is available for review by the editor of this journal.

## Conflicts of Interest

The authors declare no conflicts of interest.

## Supporting Information

Additional supporting information can be found online in the Supporting Information section.

## Supporting information


**Supporting Information 1** File S1 (CARE checklist): Completed CARE checklist for this case report.

**Supporting Information 2** File S2 (Treatment timeline): Detailed chronological timeline of diagnostic assessments, preventive/restorative care, orthodontic procedures, and follow‐up visits across the treatment period.

**Supporting Information 3** File S3 (Radiographic measurements): Complete radiographic measurement dataset and calculations (including CBCT/panoramic indices such as sector position, angulation, vertical position, and related prognostic parameters) used to assess and monitor the impacted canines.

## Data Availability

All data generated or analyzed during this case study are included in this published article. Additional supporting files are available from the corresponding author upon reasonable request.
