# Supplementary material for: Early Interceptive Management of Bilateral Canine Impaction With Anterior Crossbite Using Double Extraction and a 2 × 4 Appliance
Source: Case Rep Dent. 2026 Jul 9;2026:1080772. doi: 10.1155/crid/1080772 (PMC13347833; doi:10.1155/crid/1080772)
Supplement: Supplementary file 1 — Supporting Information 1 File S1 (CARE checklist): Completed CARE checklist for this case report. [file CRID-2026-1080772-s002.docx]

**Additional File 1: CARE Checklist**

The CARE Checklist for This Case Report

Title of Case Report: **Early interceptive management of bilateral canine impaction with anterior crossbite using double extraction and a 2×4 appliance**.

Authors: Deemah Alhamawi, BDS, SB-PD; Lena Gary, BDS, SB-Orthodontic; Raniah Baakdah, BDS, MScPD, SBPD, MME, FIAPD

The CARE (CAse REport) guidelines checklist for this case report is presented below. Each item is marked as reported (Yes/No/NA) with reference to the section or page in the manuscript where it appears.

| **#** | **CARE Checklist Item** | **Description** | **Reported (Yes/No/NA)** | **Manuscript Section/Page** |
| --- | --- | --- | --- | --- |
| 1 | Title | Identification as a case report in the title | Yes | Title page |
| 2 | Key words | 2–5 key words provided | Yes | Abstract |
| 3 | Abstract – Introduction | What is unique about this case? What is the background? | Yes | Abstract |
| 4 | Abstract – Case presentation | Main symptoms, diagnoses, interventions, and outcomes | Yes | Abstract |
| 5 | Abstract – Conclusion | Main conclusions and "take-away" lessons | Yes | Abstract |
| 6 | Introduction | Why report this case? What does it add to the literature? | Yes | Background section |
| 7 | Patient information | Age, sex, ethnicity, main presenting symptoms, relevant personal history | Yes | Case Presentation – Patient Information |
| 8 | Clinical findings | Relevant physical examination and intraoral findings | Yes | Case Presentation – Clinical Findings |
| 9 | Timeline | Important dates/time points of events, diagnosis, and treatment | Yes | Case Presentation and Additional File 2: Treatment Timeline |
| 10 | Diagnostic assessment | Diagnostic methods used, rationale, and diagnostic challenges | Yes | Case Presentation – Diagnostic Assessment section |
| 11 | Therapeutic intervention | Types of interventions, dosages, duration, and any changes in the intervention | Yes | Therapeutic Intervention section (Phase I–IV) |
| 12 | Follow-up and outcomes | Clinician and patient-reported outcomes, with specific dates | Yes | Follow-up and Outcomes section |
| 13 | Discussion | Discusses strengths, limitations, and context with literature | Yes | Discussion section |
| 14 | Patient perspective | Patient/parent perspective on condition, care, and outcomes | Yes | Follow‑up and Outcomes; Patient perspective section |
| 15 | Informed consent | Statement confirming that informed consent was obtained and documented | Yes | Declarations – Consent for Publication |

Notes:

All items are fully addressed in the manuscript according to CARE reporting guidelines.
