# Supplementary material for: Early Interceptive Management of Bilateral Canine Impaction With Anterior Crossbite Using Double Extraction and a 2 × 4 Appliance
Source: Case Rep Dent. 2026 Jul 9;2026:1080772. doi: 10.1155/crid/1080772 (PMC13347833; doi:10.1155/crid/1080772)
Supplement: Supplementary file 2 — Supporting Information 2 File S2 (Treatment timeline): Detailed chronological timeline of diagnostic assessments, preventive/restorative care, orthodontic procedures, and follow‐up visits across the treatment period. [file CRID-2026-1080772-s001.docx]

**Additional File 2: Treatment Timeline**

Detailed Chronological Sequence of All Treatment Procedures

Case: Early Detection and Treatment of Canine Impaction in Pediatrics

Patient: 9-year-old Saudi Female

Duration: Month 0 (Initial presentation) to Month 14 (Final outcome evaluation)

This file provides a comprehensive timeline of all diagnostic assessments, preventive interventions, restorative procedures, orthodontic therapy, and follow-up evaluations performed throughout the treatment course.

Treatment Timeline Table

| **Month/Week** | **Patient Age** | **Dentition Stage** | **Event/Procedure** | **Detailed Description** |
| --- | --- | --- | --- | --- |
| **Month 0** | 9 years | Early mixed | **Initial Presentation & Chief Complaint** | Patient presented to pediatric dental clinic for dental care. Chief complaint: posterior caries. Parent reported concern about anterior tooth position and spacing. |
|  |  |  | **Extraoral Examination** | Mild mandibular shift with normal TMJ function and jaw opening capacity. No facial asymmetry. No extraoral pathology noted. |
|  |  |  | **Intraoral Examination** | Poor oral hygiene noted. Multiple carious deciduous molars. MIH affecting teeth 31 (code 2) and 46 (code 3). Anterior crossbite involving teeth 11 and 12 with functional mandibular shift to the right. Absence of palpable permanent canine bulges despite normal age-expected eruption timeline. |
|  |  |  | **Baseline Radiographs: Bitewings & Periapicals** | Conventional radiographs obtained to assess caries extent and deciduous molar restorability. Established baseline for caries status and pulpal involvement. |
|  |  |  | **Baseline Radiographs: Panoramic** | Panoramic radiography revealed ectopic positioning of both maxillary permanent canines. Detailed assessment documented using Ericson–Kurol and Counihan classifications. |
|  |  |  | **Radiographic Measurements** | **Ericson & Kurol Assessment (Both Canines):** S-sector = 3 (palatal position); α-angle = 60° relative to sagittal midline; d-distance = 22 mm from canine tips to occlusal plane; V-height < half the lateral incisor root length; Apex position above first premolar roots. Classification: Bilateral palatal impaction with moderate prognosis. |
| **Month 1** | 9 years | Early mixed | **CBCT Imaging** | Three-dimensional cone-beam CT imaging performed to confirm bilateral palatal canine impaction and assess proximity to lateral incisor roots. Imaging protocol optimized for pediatric radiation exposure. Findings confirmed urgency for interceptive intervention to prevent root resorption. |
|  |  |  | **Study Models & Space Analysis** | Dental casts obtained and analyzed using Moyers space analysis. Mild maxillary crowding identified. Study models preserved for baseline documentation and future comparison. |
|  |  |  | **Phase I: Preventive Care – Oral Prophylaxis** | Comprehensive removal of plaque and calculus from all tooth surfaces. Polishing completed with fine prophylaxis paste. Bleeding on probing assessed and recorded. |
|  |  |  | **Phase I: Preventive Care – Oral Hygiene Instruction** | Personalized oral hygiene instruction provided to patient and parent. Demonstration of correct brushing technique (Bass technique), interdental cleaning with floss, and fluoride application. Compliance strategies discussed with parent. |
|  |  |  | **Phase I: Preventive Care – Dietary Counseling** | Dietary assessment and counseling regarding cariogenic foods and beverages. Parent educated on frequency of sugar consumption and recommendations for water intake. Risk-reduction strategies implemented. |
| **Month 1-2** | 9 years | Early mixed | **Phase II: Restorative Treatment – SSC on Deciduous Molars** | Stainless steel crowns (SSC) fabricated and cemented on carious deciduous molars using glass ionomer cement. Crowns selected based on tooth size, contour, and retention needs. Margins verified to ensure complete coverage and proper contact. |
|  |  |  | **Phase II: Restorative Treatment – Composite Restorations** | Composite resin restorations placed on additional carious surfaces under rubber dam isolation to maintain dry field. Composite shade matched to natural tooth color. Restorations polished and articulation verified. |
|  |  |  | **Phase II: Orthodontic Intervention – Deciduous Tooth Extraction** | Extraction of teeth 53, 54 (deciduous right canine, deciduous right first molar) and 63, 64 (deciduous left canine, deciduous left first molar): Removed to eliminate eruption obstruction and create space for spontaneous eruption of impacted canines. |
| **Month 2** | 9 years | Early mixed | **Phase II: Outcome** | Achievement of optimal oral health status with all active caries arrested and restored. Oral hygiene improved significantly. Patient and parent compliance with home care demonstrated. |
| **Month 3** | 9 years | Early mixed | **Phase III: Orthodontic Appliance – 2×4 Placement – Banding** | Maxillary first permanent molars (16, 26) were banded with size 35 bands using glass ionomer cement. Bands verified for proper fit, seating, and absence of impingement. Cement cleaned from interproximal areas. |
|  |  |  | **Phase III: Orthodontic Appliance – Bracket Bonding** | Maxillary incisor brackets bonded on teeth 12, 11, 21, 22. Enamel etched, primed, and bonded with composite adhesive. Brackets positioned according to treatment prescription. Excess composite removed and light-cured thoroughly. |
|  |  |  | **Phase III: Orthodontic Appliance – Initial Archwire** | 0.014-inch NiTi (nickel-titanium) archwire placed in all brackets and molar tubes. O-ring ligation used for secure wire engagement. Wire inserted gently to minimize initial force application. |
|  |  |  | **Phase III: Occlusal Modifications** | Glass ionomer occlusal build-ups placed on mandibular first molars (36, 46) to open vertical dimension and facilitate anterior space. Build-ups contoured for smooth occlusion and patient comfort. |
| **Week 2 post-insertion** | 9 years | Early mixed | **Archwire Progression – First Adjustment** | Upgraded to 0.016-inch NiTi archwire for increased load-deflection rate. Archwire ligated with O-rings. Patient tolerance and tooth movement assessed. |
| **Month 4-5**  **Week 5 post-insertion** | 9 years | Early mixed | **Mechanics Adjustment – Stainless Steel Archwire** | Transitioned to 0.016×0.022-inch stainless steel archwire for enhanced control. Steel ligatures applied on lateral incisors (12, 22) for precise three-dimensional control. Push coil (open coil spring) activated for midline diastema closure and incisor alignment. |
| **Month 5**  **Week 7 post-insertion** | 9 years | Early mixed | **Bracket Repositioning & Wire Adjustment** | Bracket on 11 re-bonded for improved vertical and horizontal alignment. Wire changed back to 0.016-inch NiTi for refined alignment without excessive force. |
| **Month 6** | 9 years | Mixed to early permanent | **Phase IV: Maintenance & Eruption Monitoring** | Six-monthly recall appointments scheduled. At each visit: evaluated canine eruption progress, documented occlusal development, assessed space availability, reviewed oral hygiene status. Radiographs obtained periodically to track canine movement. |
| **Month 12** | 10 years | Mixed to permanent | **Eruption Assessment** | Periapical and panoramic radiographs obtained. Canines demonstrating favorable eruptive movement in sagittal and coronal planes. No evidence of root resorption of adjacent lateral incisors. Space availability adequate for continued eruption. Appliance maintained without modification. |
| **Month 14** | ~10 years 2 months | Permanent | **Final Follow-up Evaluation – Clinical Examination** | **Bilateral Canine Eruption Status:** Both maxillary permanent canines (13, 23) erupted spontaneously into ideal occlusal positions.  **Occlusal Correction:** Complete resolution of anterior crossbite. Elimination of functional mandibular shift. Normal centric relation achieved.  **Periodontal Status:** Canines demonstrated stable periodontal attachment with healthy gingival tissues. No inflammation or probing depths.  **Adjacent Teeth:** No radiographic evidence of root resorption on lateral incisors (12, 22). Root development of canines is completing normally. |
|  |  |  | **Appliance Removal & Retention Planning** | After confirmation of canine proper positions for eruption, 2×4 appliance was removed. Brackets and bands debonded without complications. Teeth polished and cleaned. Retention plan discussed with the parent. Options for retention (removable vs. fixed vs. none) explained. |
|  |  |  | **Final Follow-up Evaluation – Radiographic Confirmation** | **Panoramic Radiograph:** Bilateral canines in ideal coronal and sagittal positions. Complete root formation progressing normally. No root resorption noted on adjacent teeth. **Periapical Radiographs:** Detailed assessment of apical anatomy and surrounding bone. Healing of extraction sites documented. No cystic lesions or apical pathology. Bone fill progressing favorably. |
| **Month 24** | 11 years | Permanent | **Post-Treatment Outcome Documentation** | Treatment duration: 14 months from extraction to complete canine eruption. **Outcomes Achieved:** ✓ Spontaneous eruption of both canines ✓ Resolution of anterior crossbite ✓ Elimination of functional shift ✓ No root resorption ✓ Stable occlusal relationships ✓ Normal function preserved ✓ Significantly improved oral hygiene ✓ Enhanced patient and parent satisfaction |

Key Treatment Milestones Summary

| **Phase** | **Duration** | **Primary Objectives Achieved** |
| --- | --- | --- |
| **Phase I – Prevention** | Months 1–2 | Optimal oral health status established; plaque control; dietary modification; risk reduction; Deciduous teeth extracted; space created |
| **Phase II – Restorative** | Months 2–4 | All active caries managed; teeth restored; foundation for orthodontic therapy secured |
| **Phase III – Orthodontic** | Months 4–14 | Fixed appliance guidance; bilateral canine eruption achieved |
| **Phase IV – Maintenance** | Ongoing post-Month 24 | Regular monitoring; occlusal stability; long-term follow-up plan established |

**Notes on Treatment Modifications**

Month 3: After initial archwire placement, patient demonstrated excellent compliance with oral hygiene despite fixed appliance. No emergency appointments for breakage or discomfort.

Week 7: Bracket repositioning on 11 was proactive measure to optimize alignment trajectory; no treatment delay resulted.

Month 10: Radiographic monitoring confirmed eruption progressing favorably; decision made to maintain appliance and continue eruption guidance rather than remove prematurely.

Follow-up Plan Beyond Month 14

Months 15–18: Quarterly recalls to monitor eruption of remaining permanent teeth and occlusal settling.

Year 2: Semi-annual appointments to assess overall development and any necessary retention/guidance.

Age 12+: Reassessment for any additional orthodontic needs as permanent dentition becomes complete.
