# Supplementary material for: Early Interceptive Management of Bilateral Canine Impaction With Anterior Crossbite Using Double Extraction and a 2 × 4 Appliance
Source: Case Rep Dent. 2026 Jul 9;2026:1080772. doi: 10.1155/crid/1080772 (PMC13347833; doi:10.1155/crid/1080772)
Supplement: Supplementary file 3 — Supporting Information 3 File S3 (Radiographic measurements): Complete radiographic measurement dataset and calculations (including CBCT/panoramic indices such as sector position, angulation, vertical position, and related prognostic parameters) used to assess and monitor the impacted canines. [file CRID-2026-1080772-s003.docx]

**Additional File 3: Radiographic Measurements**

Complete Radiographic Analysis Data and Calculations

Case: Early Detection and Treatment of Canine Impaction in Pediatrics

Patient: 9-year-old Saudi Female

Initial Assessment Date: Month 0

Final Assessment Date: Month 14-24

This file summarizes all cone-beam CT and panoramic radiographic measurements used to assess the position, severity, and prognosis of bilateral maxillary impacted canines, including Ericson and Kurol parameters and supplementary indices.

**1. Summary of Radiographic Measurements by Tooth**

**Table 1: Radiographic Assessment Parameters (Baseline – Month 0)**

| **Radiographic Parameter** | **Right Canine (13)** | **Left Canine (23)** | **Assessment Method** | **Reference Criteria** |
| --- | --- | --- | --- | --- |
| **Tooth Position** | Palatal | Palatal | CBCT & Panoramic | Buccal vs. palatal; directional displacement |
| **S-sector (Ericson & Kurol)** | 3 | 3 | Panoramic radiograph | Sector classification: 1 (mesial) to 5 (distal) relative to lateral incisor |
| **α-angle (Ericson & Kurol)** | 60° | 60° | Panoramic radiograph | Angle between canine long axis and sagittal midline; favorable <30°; unfavorable >40° |
| **d-distance (Ericson & Kurol)** | 22 mm | 22 mm | Panoramic radiograph | Distance from canine crown tip to occlusal plane; greater distance = deeper impaction |
| **Vertical Height (V-height)** | <½ LI root length | <½ LI root length | Panoramic radiograph | Vertical position relative to lateral incisor root; favorable = above root apex |
| **Apex Position** | Above first premolar roots (14, 15) | Above first premolar roots (24, 25) | CBCT | Depth of apical positioning; favorable = high, unfavorable = low/apical |
| **Buccopalatal Position** | Palatal – close to midpalatal suture | Palatal – close to midpalatal suture | CBCT (sagittal & coronal slices) | Lateral positioning in 3D space; measured from buccal plate reference |
| **Mesiodistal Angulation** | 60° to midline (mesially inclined) | 60° to midline (mesially inclined) | CBCT (coronal slice) | Angular deviation; favorable = aligned; unfavorable = severe angulation |
| **Proximity to Lateral Incisor Root (12, 22)** | Close contact, no resorption | Close contact, no resorption | CBCT (sagittal slice analysis) | Distance to adjacent root; risk of resorption if <3 mm |
| **Root Development Stage** | Apex open; ¾ root length formed | Apex open; ¾ root length formed | Panoramic radiograph | Nolla stage assessment for prognosis estimation |
| **Distance to Occlusal Plane** | 22 mm | 22 mm | Panoramic radiograph | Absolute measurement; guides eruption prognosis |

Table 2: Ericson & Kurol Classification Summary (Baseline)

| **Classification Variable** | **Right Canine (13)** | **Left Canine (23)** | **Prognosis Assessment** |
| --- | --- | --- | --- |
| **S-sector** | 3 (Palatal sector) | 3 (Palatal sector) | Moderate prognosis; palatal position requires longer eruption time compared to buccal position |
| **α-angle** | 60° | 60° | Moderate to unfavorable; angle >40° indicates increased treatment time and complexity |
| **Combined Score** | Moderate impaction severity | Moderate impaction severity | Both canines show similar moderate prognosis with reasonable potential for interceptive treatment success |

2. Supplementary Radiographic Measurements

Table 3: Three-Dimensional Analysis (CBCT – Month 0)

| **3D Measurement** | **Right Canine (13)** | **Left Canine (23)** | **Notes** |
| --- | --- | --- | --- |
| **Sagittal buccopalatal distance to buccal bone plate** | 12 mm | 12 mm | Measured on sagittal CBCT slice; indicates depth of impaction within palatal tissues |
| **Coronal mesiodistal position relative to midline** | 8 mm palatal to midline | 8 mm palatal to midline | Measured on coronal slice; symmetric bilateral positioning |
| **Axial inclination (long axis deviation)** | 55° (mesially inclined) | 55° (mesially inclined) | Measured on axial CBCT slice; relates to eruptive direction |
| **Vertical distance from apex to bone crest** | 28 mm | 28 mm | Indicates remaining eruption distance in 3D space |
| **Contact area with lateral incisor root** | ~2 mm gap | ~2 mm gap | Measured on sagittal slice at point of closest proximity; <3 mm = risk of resorption |

**3. Ericson & Kurol Measurement Definitions**

**Detailed Explanation of Key Parameters**

**S-Sector (Sector Classification):**

- Divides the maxilla into 5 sectors relative to the lateral incisor position on panoramic radiograph
- **Sector 1 (Mesial):** Canine crown distal to lateral incisor root apex; most favorable
- **Sector 2:** Canine crown overlapping with lateral incisor apical third
- **Sector 3 (Palatal):** Canine crown positioned palatally; moderate position
- **Sector 4:** Canine crown positioned further palatally; less favorable
- **Sector 5 (Distal):** Canine crown far distal; unfavorable position
- **This case:** S-sector 3 (palatal) indicates moderate impaction requiring intervention

**α-angle (Alpha Angle):**

- Angle measured between the long axis of the impacted canine and the mid-sagittal plane
- **Measurement method:** Panoramic radiograph; bisect angle of canine long axis and vertical midline
- **Favorable:** <30° (canine more aligned with midline)
- **Moderate:** 30°–40° (moderate angulation)
- **Unfavorable:** >40° (severe angulation; longer treatment time; lower success rates)
- **This case:** α-angle 60° indicates significant mesial inclination requiring correction; unfavorable for interceptive treatment alone

**d-distance (Distance from Cusp Tip to Occlusal Plane):**

- Linear measurement from canine crown cusp tip to maxillary occlusal plane
- **Measurement method:** Perpendicular line from cusp to occlusal plane on panoramic radiograph
- **Favorable:** <7 mm (shallow impaction; high eruption potential)
- **Moderate:** 7–13 mm (intermediate depth; reasonable prognosis)
- **Unfavorable:** >13 mm (deep impaction; lower eruption potential)
- **This case:** d-distance 22 mm indicates deep impaction; unfavorable factor requiring significant space creation

**V-height (Vertical Height):**

- Vertical position of canine crown relative to lateral incisor root length
- **Measurement method:** Compare vertical extent of canine crown to root length of adjacent lateral incisor
- **Favorable:** Crown level above apical third of adjacent root
- **Moderate:** Crown level at apical third to apical half of root
- **Unfavorable:** Crown level below apical half of root (<½ lateral incisor root length)
- **This case:** V-height <½ lateral incisor root length; unfavorable indicator

**4. Radiographic Changes Over Treatment Course**

**Table 4: Canine Position Assessment at Key Timepoints**

| **Assessment Timepoint** | **d-distance (Right 13)** | **d-distance (Left 23)** | **S-sector Position (Both)** | **Proximity to Lateral Incisor Root** | **Notes** |
| --- | --- | --- | --- | --- | --- |
| **Month 0 (Baseline)** | 22 mm | 22 mm | Sector 3 (palatal) | ~2 mm gap; no resorption | Deep impaction; both canines at equivalent levels |
| **Month 2** | 20 mm | 20 mm | Sector 3, shifting buccal | ~3 mm gap | Early response to space creation; slight movement noted |
| **Month 6** | 16 mm | 16 mm | Sector 2/3 transition | ~4 mm gap; no resorption | Steady eruptive movement; favorable trajectory |
| **Month 10** | 10 mm | 10 mm | Sector 2 (buccal-shifting) | ~5 mm gap; no resorption | Accelerated eruption; approaching occlusal plane |
| **Month 14 (Final)** | 0 mm (erupted) | 0 mm (erupted) | Sector 1 (fully erupted into ideal position) | Normal contact; no resorption | Complete eruption; occlusal contact established |
| **Change from Baseline to Final (Δ)** | **−22 mm** | **−22 mm** | **Sector 3 → Sector 1** | **Maintained gap; resorption-free** | **Complete successful eruption** |

5. Root Development and Apex Assessment

Table 5: Root Development Staging (Nolla Classification)

| **Assessment Date** | **Nolla Stage (Right 13)** | **Nolla Stage (Left 23)** | **Root Development Progress** |
| --- | --- | --- | --- |
| **Month 0 (Baseline)** | Stage 6/7 (¾ root length formed; apex open) | Stage 6/7 (¾ root length formed; apex open) | Approximately 75% of final root length developed; apex still widely patent |
| **Month 6** | Stage 7/8 (apical third forming) | Stage 7/8 (apical third forming) | Continued root development during eruptive movement |
| **Month 14 (Final)** | Stage 8/9 (root nearly complete; apex nearly closed) | Stage 8/9 (root nearly complete; apex nearly closed) | Root development continuing normally during/after eruption; no arrest or acceleration |

6. Adjacent Tooth Assessment (Lateral Incisors 12 and 22)

Table 6: Lateral Incisor Root Integrity During Treatment

| **Assessment Date** | **Root Length 12** | **Root Resorption 12** | **Root Length 22** | **Root Resorption 22** | **Bone Level Adjacent to 12 and 22** |
| --- | --- | --- | --- | --- | --- |
| **Month 0 (Baseline)** | Normal (~14 mm) | None visible | Normal (~14 mm) | None visible | Normal crest level |
| **Month 6** | ~14 mm (unchanged) | None detected | ~14 mm (unchanged) | None detected | Normal, maintained |
| **Month 14 (Final)** | ~14 mm (maintained) | None (0%) | ~14 mm (maintained) | None (0%) | Normal, no defects |
| **Conclusion** | **No resorption despite proximity** | **FAVORABLE** | **No resorption despite proximity** | **FAVORABLE** | **Healthy periodontium maintained** |

7. Bone Remodeling and Space Availability

Table 7: Alveolar Bone Assessment

| **Aspect** | **Baseline (Month 0)** | **Mid-treatment (Month 6)** | **Final (Month 24)** | **Interpretation** |
| --- | --- | --- | --- | --- |
| **Extraction site healing (53, 63)** | N/A – intact | Early bone fill; socket defined | Complete bone fill; canine space available | Excellent healing; adequate bone density for eruption guidance |
| **Extraction site healing (54, 64)** | N/A – intact | Bony trabeculae filling | Mature bone; sclerotic margins | Complete healing; space maintained |
| **Maxillary bone density** | Normal | Normal; no pathology | Normal; no complications | No adverse bone changes during treatment |
| **Periapical bone around canine apices** | Normal; clear lamina dura | Normal throughout | Normal; apices approaching completion | Healthy periapical tissues; no infections |

**8. Radiographic Measurements: Calculations and Interpretations**

**Eruption Velocity Calculation**

**Eruption rate (mm/month) = Change in d-distance ÷ Number of months**

- **Baseline d-distance (Month 0):** 22 mm
- **Final d-distance (Month 24):** 0 mm
- **Total movement:** 22 mm
- **Time elapsed:** 14 months
- **Eruption velocity:** 22 mm ÷ 14 months = **1.57 mm/month**

**Interpretation:** Eruption velocity of 1.57 mm/month is within normal range for guided eruption of impacted canines following deciduous tooth extraction and space creation. Literature suggests typical rates of 1–3 mm/month for successfully guided impacted canines.

**9. Prognostic Factors Analysis**

**Table 8: Factors Influencing Treatment Success**

| **Prognostic Factor** | **Baseline Status** | **Impact on Prognosis** | **Outcome at Month 14** |
| --- | --- | --- | --- |
| **Age at treatment** | 9 years old | Favorable – mixed dentition; high skeletal growth potential | Positive – utilized growth period effectively |
| **Impaction depth (d-distance)** | 22 mm (deep) | Moderate to unfavorable – significant space needed | Overcome by deciduous tooth extraction and appliance guidance |
| **Canine inclination (α-angle)** | 60° (mesially inclined) | Unfavorable – requires correction | Corrected by fixed appliance mechanics |
| **S-sector position** | Sector 3 (palatal) | Moderate – palatal position requires longer eruption time | Successfully guided to buccal (Sector 1) position |
| **Lateral incisor proximity** | 2 mm gap (close) | Moderate risk of resorption | **Successfully prevented** – no resorption at final evaluation |
| **Root development stage** | ¾ root length formed | Favorable – allows directional control during eruption | Root completed normally in ideal eruption path |
| **Patient age at extraction** | 9 years | Favorable – greater remodeling potential | Excellent space maintenance and healing |
| **Patient compliance** | Excellent (demonstrated) | Excellent compliance essential for success | High compliance maintained throughout treatment |

**10. Radiographic Findings Summary**

**Key Findings Documentation**

**Baseline Assessment (Month 0):**

- Bilateral maxillary canine impaction (palatal position, Sectors 3)
- Moderate impaction severity per Ericson & Kurol criteria
- d-distance: 22 mm (deep); α-angle: 60° (mesially inclined)
- Close proximity to lateral incisor roots without baseline resorption
- Adequate bone volume and bone density for eruption guidance
- Anterior crossbite with functional mandibular shift

**Progressive Assessment (Month 6):**

- Favorable eruptive movement in both canines (~6 mm per canine)
- Gradual transition from palatal to buccal position (Sector 3→2)
- Extraction sites healing with mature bone fill
- No resorption developing on adjacent lateral incisor roots
- Crossbite correction progressing; incisor alignment improving

**Final Assessment (Month 24):**

- Complete bilateral eruption into ideal occlusal positions
- **Right canine (13):** Fully erupted; Sector 1 position; normal crown-root anatomy
- **Left canine (23):** Fully erupted; Sector 1 position; normal crown-root anatomy
- Root development completing normally (Nolla Stage 8/9)
- **Zero resorption** of adjacent lateral incisor roots (12, 22) maintained throughout
- Anterior crossbite resolved; normal incisor relationship achieved
- Functional mandibular shift eliminated
- Alveolar bone remodeling complete; healthy periodontal tissues

**11. References for Measurement Methods**

1. **Ericson S, Kurol J.** (1988). Longitudinal study and analysis of clinical supervision of maxillary canine eruption. *Community Dent Oral Epidemiol.* 16:172–176.
2. **Counihan K, Al-Awadhi EA, Butler J.** (2012). Guidelines for the assessment of the impacted maxillary canine. *Dent Update.* 40:770–777.
3. **Nolla CM.** (1960). The development of the permanent teeth. *J Dent Child.* 27:254–266.
4. **Becker A, Chaushu S.** (2023). Etiology of maxillary canine impaction: comprehensive review of current evidence. *Am J Orthod Dentofacial Orthop.* 163:445–458.

**12. Conclusion**

Radiographic measurements document the successful resolution of bilateral palatal maxillary canine impaction through early detection, interceptive deciduous tooth extraction, and controlled orthodontic guidance. The absence of root resorption, coupled with complete eruption into ideal positions within 14 months, demonstrates the efficacy of the treatment protocol and optimal timing of intervention during the mixed dentition stage.

**All radiographic data support a successful, complication-free treatment outcome.**
